# Cytokine hemoadsorption with CytoSorb^®^ in post-cardiac arrest syndrome, a pilot randomized controlled trial

**DOI:** 10.1186/s13054-023-04323-x

**Published:** 2023-01-23

**Authors:** Céline Monard, Nathan Bianchi, Elettra Poli, Marco Altarelli, Anne Debonneville, Mauro Oddo, Lucas Liaudet, Antoine Schneider

**Affiliations:** 1 Adult Intensive Care Unit, Centre Hospitalier Universitaire Vaudois (CHUV), Lausanne, Switzerland; 2 Thoracic Surgery Unit, Centre Hospitalier Universitaire Vaudois (CHUV), Lausanne, Switzerland; 3 Faculty of Biology and Medicine (FBM), University of Lausanne (UNIL), Lausanne, Switzerland; 4 Medical Directorate, Centre Hospitalier Universitaire Vaudois (CHUV), Lausanne, Switzerland

**Keywords:** Cardiac arrest, Cytokine hemoadsorption, Extracorporeal blood purification, Hemoperfusion, Inflammation, Post-cardiac arrest syndrome

## Abstract

**Background:**

Hemoadsorption (HA) might mitigate the systemic inflammatory response associated with post-cardiac arrest syndrome (PCAS) and improve outcomes. Here, we investigated the feasibility, safety and efficacy of HA with CytoSorb^®^ in cardiac arrest (CA) survivors at risk of PCAS.

**Methods:**

In this pilot randomized controlled trial, we included patients admitted to our intensive care unit following CA and likely to develop PCAS: required norepinephrine (> 0.2 µg/kg/min), and/or had serum lactate > 6 mmol/l and/or a time-to-return of spontaneous circulation (ROSC) > 25 min. Those requiring ECMO or renal replacement therapy were excluded. Eligible patients were randomly allocated to either receive standard of care (SOC) or SOC plus HA. Hemoadsorption was performed as stand-alone therapy for 24 h, using CytoSorb^®^ and regional heparin–protamine anticoagulation. We collected feasibility, safety and clinical data as well as serial plasma cytokines levels within 72 h of randomization.

**Results:**

We enrolled 21 patients, of whom 16 (76%) had out-of-hospital CA. Median (IQR) time-to-ROSC was 30 (20, 45) minutes. Ten were assigned to the HA group and 11 to the SOC group. Hemoadsorption was initiated in all patients allocated to the HA group within 18 (11, 23) h of ICU admission and conducted for a median duration of 21 (14, 24) h. The intervention was well tolerated except for a trend for a higher rate of aPTT elevation (5 (50%) vs 2 (18%) *p* = 0.18) and mild (100–150 G/L) thrombocytopenia at day 1 (5 (50%) vs 2 (18%) *p* = 0.18). Interleukin (IL)-6 plasma levels at randomization were low (< 100 pg/mL) in 10 (48%) patients and elevated (> 1000 pg/mL) in 6 (29%). The median relative reduction in IL-6 at 48 h was 75% (60, 94) in the HA group versus 5% (− 47, 70) in the SOC group (*p* = 0.06).

**Conclusions:**

In CA survivors at risk of PCAS, HA was feasible, safe and was associated with a nonsignificant reduction in cytokine plasma levels. Future trials are needed to further define the role of HA after CA. Those studies should include cytokine assessment to enrich the study population.

*Trial registration*: NCT03523039, registered 14 May 2018.

**Supplementary Information:**

The online version contains supplementary material available at 10.1186/s13054-023-04323-x.

## Background

Cardiac arrest (CA) occurs in close to 2.0 per 1000 person-years in Europe and is associated with high morbidity and mortality [1]. Among patients successfully resuscitated and admitted to the intensive care unit (ICU), only 40% are discharged alive and a small portion of those ultimately returns home [2, 3].

A significant proportion of in-hospital deaths may be attributable to post-cardiac arrest syndrome (PCAS) and its systemic consequences [4]. PCAS develops in the hours (6–24) following the return of spontaneous circulation (ROSC) and is characterized by multiple organ dysfunctions including hemodynamic failure, myocardial injury and neurological damage [5].

To date, despite its major clinical relevance, there is no specific therapy for PCAS and its management is only supportive [6]. However, new understanding of PCAS pathophysiology may create opportunities for therapeutic actions. Indeed, PCAS is thought to be related to global ischemia–reperfusion injury and is associated with high blood levels of pro- and anti-inflammatory cytokines, including interleukins (IL)-6, IL-8, IL-10, and tumor necrosis factor (TNF)-α [7]. These cytokines, particularly IL-6, may play a role in organ damage through endothelial dysfunction, vascular leakage and vasodilation [7]. Elevated IL-6 at admission, in the blood or in the cerebrospinal fluid, is associated with mortality after CA and poor neurological outcomes [8–11]. Hence, early reduction in blood cytokine levels with extracorporeal blood purification therapies might mitigate the inflammatory response, decrease the extent of PCAS and lead to improved outcomes [12]. Such cytokine level reduction was previously evaluated with high-volume hemofiltration and was associated with a decreased risk of death from intractable shock [13]. However, high-volume hemofiltration is notoriously difficult to deliver and other techniques might be more applicable at the bedside [14]. The CytoSorb^®^ hemoadsorption cartridge has been designed to remove cytokines and can be used as stand-alone therapy or in combination with other extracorporeal organ support therapies such as renal replacement therapy (RRT) or extracorporeal membrane oxygenation (ECMO). To date, there is no evaluation of stand-alone HA with CytoSorb^®^ in CA survivors at risk of PCAS and without ECMO.

The aim of this study was to evaluate the feasibility, safety and efficacy of hemoadsorption with CytoSorb^®^ in cardiac arrest survivors admitted to the intensive care unit (ICU) and at risk of PCAS.

## Methods

### Study design and population

This pilot single-center open-label randomized controlled trial was conducted in the Centre Hospitalier Universitaire Vaudois (CHUV), Lausanne, Switzerland. We considered for inclusion all adult patients admitted to our institution’s intensive care unit (ICU) following in- or out-of-hospital cardiac arrest. Those who presented one of the following criteria within 24 h of admission were eligible: time-to-ROSC > 25 min and/or serum lactate level > 6 mmol/l and/or need for norepinephrine > 0.2 µg/kg/min for ≥ 1 h to maintain mean arterial pressure (MAP) > 60–70 mmHg. In the absence of a consensual definition for PCAS, these criteria were chosen based on their association with IL-6 plasma levels or post-cardiac arrest shock, a major feature of PCAS accounting for most deaths within the first 3 days [15, 16]. Exclusion criteria included evidence of the patient’s refusal to participate in clinical trials, imminent withdrawal of care, pregnancy, refractory CA with ECMO implantation, need for RRT at the time of randomization, active bleeding or high risk of bleeding.

Since eligible patients were expected to be unable to provide informed consent prior to randomization and since the intervention could not be delayed, an emergency consent procedure was performed. According to this procedure, patients without documented refusal to participate in clinical trials could be included pending the approval of a physician independent from the trial. Consent from a proxy was sought as soon as possible as well as from the patient himself once able to provide informed consent. The study protocol was approved by the Ethics Committee of Vaud (2018-00421) and registered at ClinicalTrials.gov (NCT03523039).

### Intervention

Patients were randomized on a 1:1 basis to receive either standard of care (SOC) or standard of care plus hemoadsorption (HA). Randomization sequence was generated using the Excel (Microsoft, Redmond, USA) Rand () function with a 1:1 ratio and permuted blocks of variable sizes. The allocation group was stored within numbered sealed envelopes.

All patients were managed according to local protocol and international guidelines. This included 24 h of targeted temperature management with a central temperature target at 35–36 °C, hemodynamic management targeting a mean arterial pressure > 70 mmHg, glycemic control, avoidance of hyperoxia (PaO_2_ target 70–100 mmHg), and sedation.

For patients allocated to the intervention group, HA was performed as a stand-alone therapy, using a CytoSorb^®^ cartridge (CytoSorbents Corporation, Monmouth Junction, NJ, USA) and a multiFiltrate^®^ monitor (Fresenius Medical Care, Bad Homburg, Germany) in hemoperfusion mode. Blood flow was maintained > 200 ml/min, and ideally between 250 and 400 ml/min. In order to minimize the risk of bleeding, regional circuit anticoagulation was achieved with a heparin–protamine regimen (see Additional file 1) [17]. After the insertion of a dialysis catheter in a femoral vein, the therapy was initiated within 6 h of randomization and maintained for a minimum of 12 h and up to 24 h. Patients with early (< 12 h) circuit failure, were not included in the efficacy analysis. At the end of the intervention, the dialysis catheter was removed and sent to the laboratory for microbiological analysis.

### Endpoints

Since this was a pilot study, we assessed the procedure’s feasibility, safety and efficacy.

Feasibility was assessed by the ratio of included/screened patients, the time to therapy initiation and the percentage of patients who actually received > 12 h of HA in the intervention group.

Safety endpoints included anaphylactoid reactions, bronchospasm, bleeding complications, need for red blood cells transfusion, coagulation abnormalities (defined as a systemic aPTT ≥ 1.5 times baseline value), catheter-related complications (thrombosis, hematoma at insertion site, infections) and new-onset thrombopenia (thrombocytes < 150 G/l) within one and 2 days after randomization. Any other untoward event was recorded and reported.

Efficacy was assessed by the trend in cytokines’ plasma levels within 72 h of randomization and their absolute and relative reduction at 48 h compared to the baseline value (randomization). A large panel of cytokines was assessed including pro-inflammatory (IL-1β, IL-2, IL-6, IL-8, IL-5, interferon (IFN)-γ, GM-CSF, TNF-α) and anti-inflammatory (IL-4, IL-10) mediators. The relative reduction was calculated using the following formula:

$$\begin{aligned} {\text{Relative}}\, {\text{reduction}}\, {\text{at}} 48\,{\text{hours}}\, = & \,\frac{{{\text{absolute}} \,{\text{reduction}} \,{\text{at}} \,48 \,{\text{hours}}}}{{{\text{value}} \,{\text{at}} \,{\text{randomization}} }} \\ \, = & \, \frac{{{\text{value}} \,{\text{at}} \,{\text{randomization}} - {\text{value}}\,{\text{at}}\,48\, {\text{hours}}}}{{{\text{value}} \,{\text{at}} \,{\text{randomization}} }} \\ \end{aligned}$$

Blood samples for cytokines analysis were drawn from the arterial line or the central venous catheter at six time points (randomization, 6, 12, 24, 48 and 72 h post-randomization) into ethylenediaminetetraacetic acid tubes. They were centrifuged at 1500 RPM at 4 °C for 10 min. Three aliquots of 100 μl plasma were prepared and stored at − 80 °C. Cytokines analyses were performed using a Human Cytokine Magnetic 10-Plex Panel on the Luminex^®^ Platform (Thermo Fischer Scientific^®^, Waltham, USA). For IL-6, an enzyme-linked immunosorbent assay (ELISA) analysis was also performed. All analyses were conducted according to the manufacturer’s protocol and results are reported in pg/mL.

Secondary endpoints included serum levels of C-reactive protein at day 2, acute kidney injury (AKI) during ICU stay (as defined by the Kidney Disease Improving Global Outcomes (KDIGO) guidelines 2012 [18]), change in SOFA score at day 1 and 2, all-cause mortality at day-14 and day-28, ICU length of stay (LOS), 50% decrease in vasopressors dose within 24 and 48 h and shock reversal within 24 h. Shock reversal was defined as a sustained decrease in norepinephrine requirement to < 10 µg/min and a sustained normalization of serum lactate level (< 2 mmol/l).

### Statistical analyses

For this pilot study, a convenience sample of 20 patients per arm was arbitrarily decided. The protocol allowed to decrease this sample size to 10 patients per arm for either safety reasons or slow recruitment. Patients with an intervention duration < 12 h were excluded from the efficacy analysis.

All data were collected and stored within a secured database. Qualitative variables are expressed as numbers (percentages) and continuous variables as median (interquartile range—IQR). Due to the exploratory character of the study and the small number of patients, we only performed descriptive analyses with simple comparison tests between groups. Comparisons of secondary outcomes were made using Fisher’s exact test or Wilcoxon rank-sum test, as appropriate, and were exploratory. In the efficacy analysis, we considered only cytokines with > 50% of values above detection thresholds at randomization and at 48 h. A two-tailed *p *value < 0.05 was considered statistically significant. Analyses were performed with STATA version 17.0 (StataCorp LP, College Station, Texas) and figures created with GraphPad Prism 7 (GraphPad Software, USA).

## Results

### Participants’ characteristics

**Fig. 1:**
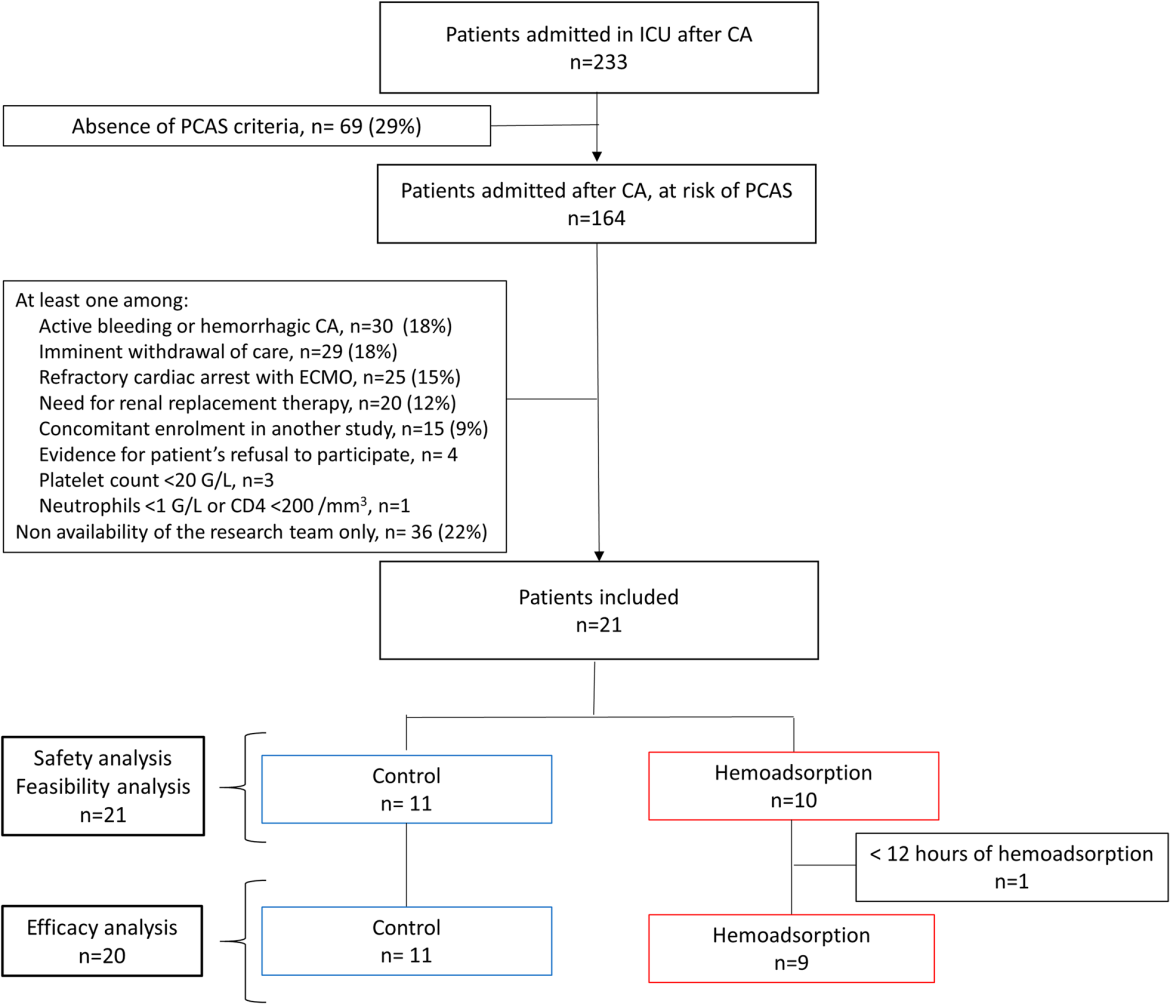
Flowchart of participants. CA cardiac arrest, ECMO extracorporeal membrane oxygenation, ICU intensive care unit, PCAS post-cardiac arrest syndrome. PCAS is defined by at least one criterion among a time-to-ROSC > 25 min and/or a serum lactate level > 6 mmol/l and/or > 1 h of norepinephrine infusion > 0.2 µg/kg/min (or equivalent vasoconstrictor agent) to maintain mean arterial pressure (MAP) > 60–70 mmHg

Between February 2019 and August 2021, 233 patients were admitted following CA to our ICU, including 164 (71%) with at least one criterion for PCAS. Among the reasons for exclusion, the main ones were ECMO or RRT initiation (*n* = 45, 27%), non-availability of the research team (*n* = 36, 22%), contraindication to heparin anticoagulation (*n* = 30, 18%) and imminent withdrawal of care (*n* = 29, 18%). Hence, 21 patients were included and randomized to receive HA (*n* = 10) or standard of care (*n* = 11) (Fig. 1). One patient in the intervention group received less than 12 h of hemoadsorption and was included only in the feasibility and safety analysis. The study was interrupted after the inclusion of 21 patients because of major delays in inclusions due to the suspension of clinical trials during the COVID-19 pandemia.

Patients’ baseline characteristics are presented in Table 1. The proportion of patients with out-of-hospital CA and shockable rhythm was higher in the intervention group compared with the control group (Table 1). Median low-flow duration was 20 (15, 40) min in the control group versus 30 (22, 45) minutes in the HA group. The median time between CA and randomization was 13 (8, 17) h.

**Table 1:** Baseline characteristics of participants

	Control (*n* = 11)	Hemoadsorption (*n* = 10)	*p* value
Patients’ characteristics			
Age, years	68 (48, 72)	72 (63, 77)	0.16
Charlson score	5 (1, 5)	5 (3, 6)	0.33
Chronic kidney disease (GFR < 30 ml/min)	1 (9%)	1 (10%)	1.00
Chronic heart failure	1 (9%)	4 (40%)	0.15
Diabetes	3 (27%)	3 (30%)	1.00
Hypertension	9 (82%)	4 (40%)	0.08
COPD	2 (18%)	1 (10%)	1.00
Active smoker	9 (82%)	5 (50%)	0.18
Aspirin	8 (73%)	2 (20%)	0.03
Blockers of the renin–angiotensin system	5 (45%)	3 (30%)	
Cardiac arrest characteristics			
No-flow duration, minutes	4 (0, 9)	4 (1, 10)	0.81
Low-flow duration, minutes	20 (15, 40)	30 (22, 45)	0.31
Time-to-ROSC	22 (18, 40)	36 (24, 50)	0.24
Presenting rhythm			0.08
Non-shockable	7 (64%)	2 (20%)	
Shockable	4 (36%)	8 (80%)	
Cause of cardiac arrest			1.00
Ischemic	5 (45%)	6 (60%)	
Hypoxic	2 (18%)	1 (10%)	
Other	4 (37%)	3 (30%)	
Location of cardiac arrest			0.31
In-hospital	4 (36%)	1 (10%)	
Out-of-hospital	7 (64%)	9 (90%)	
Laboratory at ICU admission			
Serum creatinine, µmol/L	117 (92, 204)	133 (123, 210)	0.22
Lactate, mmol/L	7 (5, 12)	4 (3, 5)	0.02
Hemoglobin, g/L	122 (109, 137)	130 (120, 149)	0.44
C-reactive protein, mg/L	5 (2, 18)	3 (2, 70)	0.96
Thrombocytes, G/l	192 (111, 225)	203 (172, 261)	0.42
aPTT, sec	34 (30, 39)	41 (34, 150)	0.16
Plasma interleukin-6, pg/mL (at randomization)	79 (55, 11,296)	319 (80, 19,653)	0.40
Scores and vasopressors at randomization			
SOFA score	12 (9, 13)	11 (8, 13)	0.52
Noradrenalin, µg/min	10 (4, 20)	16 (6, 32)	0.60
Targeted temperature management			
Delay ICU admission to TTM, hours	3 (0.5, 4)	4 (2, 5)	0.45
Delay TTM to randomization, hours	10 (− 0.5, 12)	8 (0.25, 17)	0.48
Average temperature during TTM, °C	35 (35, 36)	35 (35, 36)	0.46
Duration of TTM, hours	23 (17, 24)	24 (22, 24)	0.43

Data are presented as median (interquartile range—IQR) for continuous measures, and *n* (%) for categorical measures. Continuous measures are compared with a Wilcoxon rank-sum test and categorical measures are compared with a Fisher's exact test

*aPTT* activated partial thromboplastin clotting time, *COPD* chronic obstructive pulmonary disease, *GFR* glomerular filtration rate, *ICU* intensive care unit, *ROSC* return of spontaneous circulation, *TTM* targeted temperature management

Median IL-6 plasma level at randomization was nonsignificantly higher in the HA group compared with the control group (319 (80, 19,654) vs 79 (55, 11,296) pg/ml (*p* = 0.4)).

### Feasibility and safety

HA could be initiated in all patients randomized to the intervention group within a median delay of 25 (20, 27) h after CA, 18 (11, 23) hours after ICU admission and 3 (3, 5) h after randomization. The treatment median duration was 21 (14, 24) h. Other therapy characteristics are presented in the Additional file 2. HA was interrupted after 13 h in two patients; one because of withdrawal of medical care due to reconsideration of the therapeutic plan and one because of urgent RRT initiation. One patient received HA for less than 12 h because of excessive heparin administration due to syringe pump dysfunction. The patient was excluded from the efficacy analysis, and this event was reported as a serious adverse event.

The intervention was associated with an elevated systemic aPTT in 5 patients in the intervention group. Similarly, it was associated with a trend toward a higher rate of new-onset thrombopenia (< 150 G/L) within 2 days (5 (56%) vs 3 (30%), *p* = 0.37). The intervention was not associated with a higher need for red blood cells (RBC) transfusions within 2 days of randomization. Clinically relevant bleeding was observed in one patient in the intervention group (hemoptysia) and one in the control group (upper gastrointestinal bleeding), both on randomization day. After medical review, the complication was not attributed to the procedure. No other adverse events were observed, particularly no catheter-related complications such as infection, hematoma or thrombosis.

### Efficacy

**Fig. 2:**
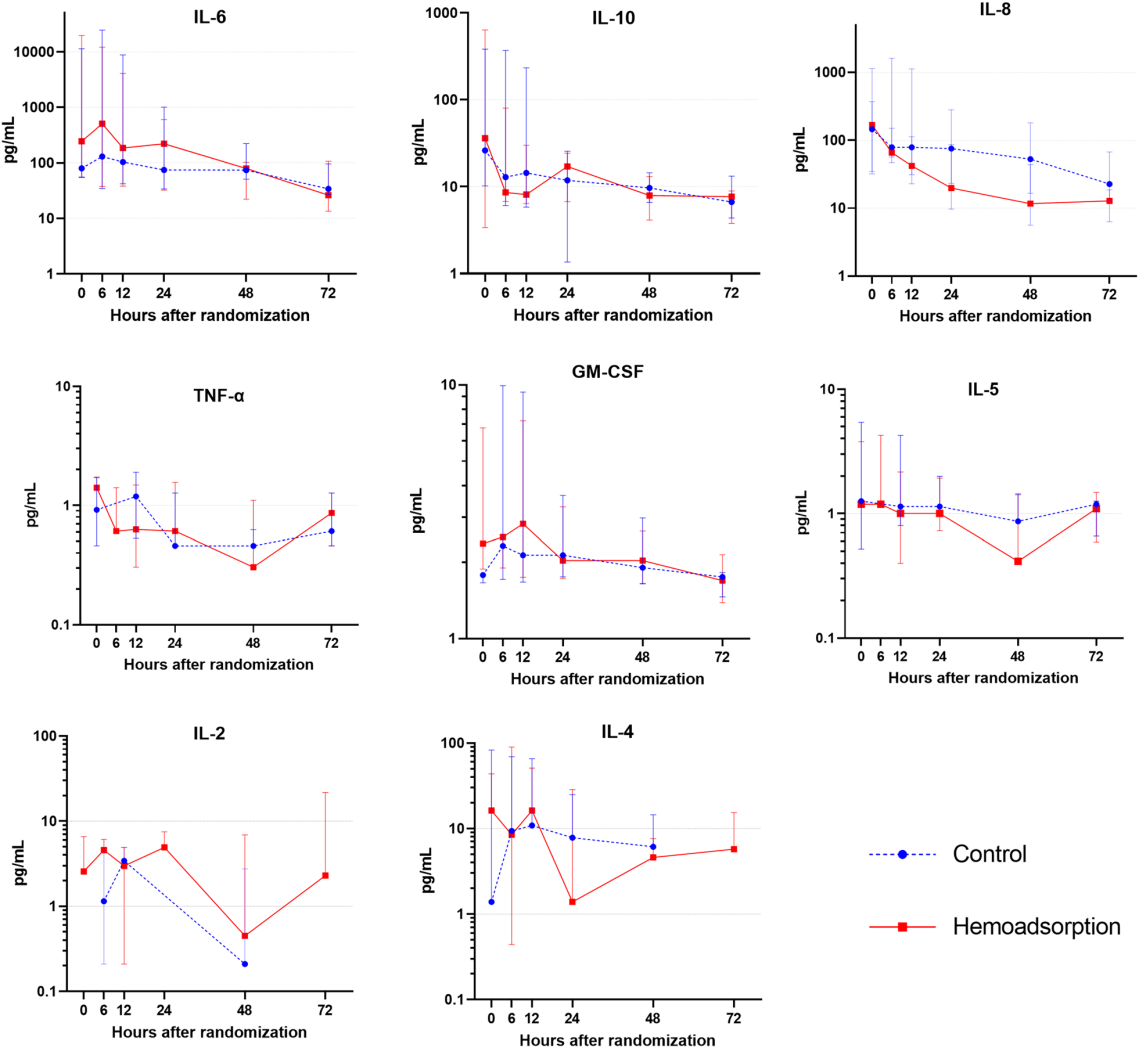
Plasma cytokines levels within 72 h of randomization. Median and interquartile range for cytokine plasma levels in the control group and in the hemoadsorption group, within 72 h of randomization. H_0_ randomization, IL interleukin, IFN-γ interferon-gamma, TNF-α tumor necrosis factor-alpha (*n* = 20 at H0, H6, H12, H24; *n* = 18 at H48 and *n* = 11 at H72)

Plasma levels of IL-1β and IFN-γ were below detection thresholds in more than 50% of the patients at randomization and at 48 h, and these cytokines were not considered for analyses. Trends and median values in cytokine plasma levels within 72 h of randomization are reported in Fig. 2.

Overall, we observed a downward trend for plasma levels of all cytokines throughout the 72 h post-randomization. 48 h after randomization, the median decrease in IL-6 plasma levels, relative to baseline, was 75% (60, 94) in the HA group versus 5% (− 47, 70) in the control group (*p* = 0.06).

In cytokines with low median plasma levels at baseline (< 10 pg/mL) including TNF-α, GM-CSF, IL-5, IL-2, and IL-4, absolute decreases at 48 h were small, but accounting for baseline values, a nonsignificant higher relative reduction was observed in the HA group versus the control group (Table 2).

**Table 2:** Efficacy outcome: reduction in cytokines plasma levels within 48 h post-randomization

	Control (*n* = 10*)	Hemoadsorption (*n* = 8*)	Total (*n* = 18)	*p* value
*IL-6*				
Absolute reduction, pg/mL	4.18 (− 13, 639)	143 (42, 19,555)	43 (− 7, 792)	0.38
Relative reduction, %	5 (− 47, 7)	75 (60, 94)	60 (− 13, 86)	0.06
*IL-8*				
Absolute reduction, pg/mL	59 (10, 187)	181 (21, 345)	128 (16, 266)	0.37
Relative reduction, %	72 (13, 90)	90 (68, 98)	83 (33, 93)	0.06
*IL-10*				
Absolute reduction, pg/mL	15 (4, 140)	82 (0.15, 617)	20 (2, 366)	0.59
Relative reduction, %	62 (41, 76)	91 (35, 99)	75 (41, 92)	0.20
*TNF-α*				
Absolute reduction, pg/mL	0.15 (− 0.46, 1.71)	1.35 (− 0.25, 1.56)	0.84 (− 0.46, 1.56)	0.72
Relative reduction, %	60 (5, 100)	100 (96, 100)	98 (25, 100)	0.29
*IL-5*				
Absolute reduction, pg/mL	0.12 (− 0.41, 0.83)	1.35 (0.09, 3.00)	0.78 (− 0.41, 1.51)	0.25
Relative reduction, %	20 (2, 100)	100 (89, 100)	94 (4, 100)	0.10
*IL-2*				
Absolute reduction, pg/mL	0.00 (0.00, 3.58)	3.47 (− 2.63, 6.59)	0.00 (0.00, 5.26)	0.39
Relative reduction, %	77 (35, 90)	82 (63, 100)	77 (63, 100)	0.67
*IL-4*				
Absolute reduction, pg/mL	0.00 (− 1.60, 1.39)	12.35 (1.20, 37.97)	0.69 (− 1.39, 13.57)	0.07
Relative reduction, %	38 (− 14, 93)	84 (72, 97)	75 (16, 97)	0.47
*GM-CSF*				
Absolute reduction, pg/mL	0.04 (− 0.35, 0.35)	0.65 (0.02, 3.86)	0.13 (− 0.21, 1.20)	0.16
Relative reduction, %	2 (− 14, 16)	27 (2, 49)	8 (− 14, 31)	0.16

Data are presented as median (interquartile range—IQR), and compared between groups with Fisher exact test

Absolute reduction is calculated as: (value at randomization - value at 48 h)

Relative reduction is calculated as the ratio of the absolute reduction over the value at randomization. This ratio could not be calculated when value at randomization was under detection threshold

*One patient in each group died within 48 h, reduction at 48 h could be evaluated in 10 patients in the control group and 8 patients in the hemoadsorption group

**Fig. 3:**
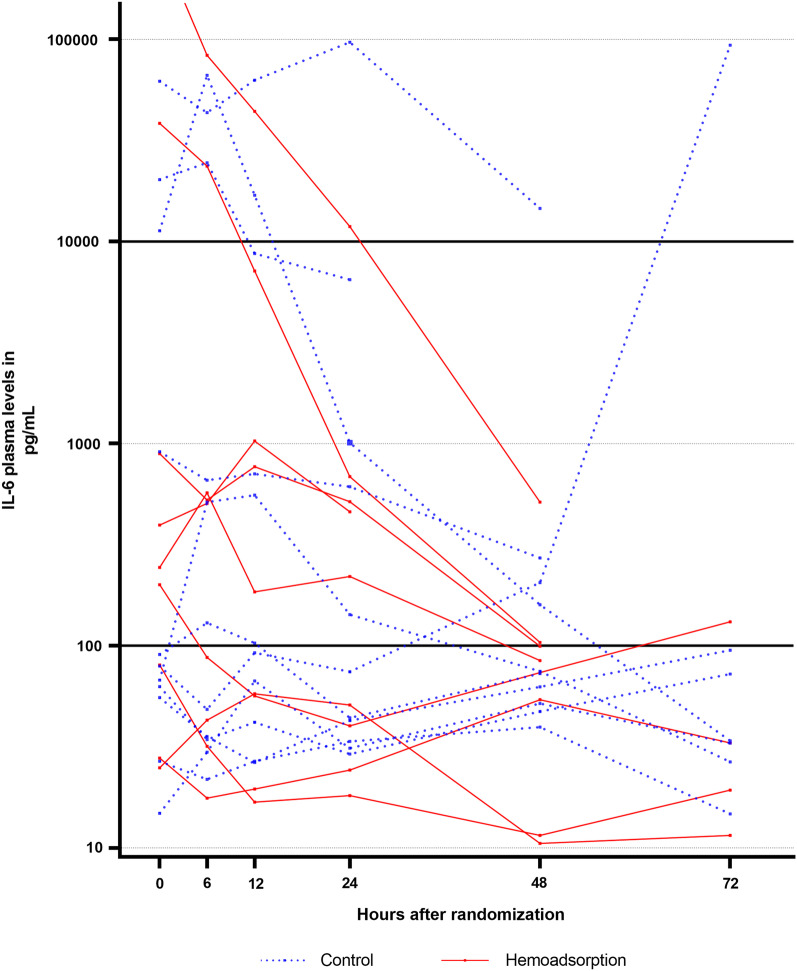
Individual trajectories of Interleukin-6 plasma levels within 72 h of randomization. Individual trajectories of Interleukin-6 plasma levels within 72 h of randomization (*n* = 20)

Among patients with very high IL-6 level at randomization (> 10,000 pg/mL, *n* = 5), 2/2 patients in the HA group decreased their level to < 1000 pg/mL within 48 h versus only 1/3 in the control group (Fig. 3).

There was no correlation between IL-6 plasma levels at randomization and time-to-ROSC, location of cardiac arrest (in- or out-of-hospital), presenting rhythm, lactate at admission or randomization, PCT at admission and CRP at admission (Pearson’s correlation coefficient < 0.6 for each).

### Secondary endpoints

There was no difference between the two groups in terms of ICU length of stay or survival at day 28. Of note, all deaths occurred in the ICU, before day 8. The cause of death was the withdrawal of life support due to irreversible brain damage in 11/14 (79%) patients. There was a greater hemodynamic improvement at 48 h in the control group than in the HA group (Table 3).

**Table 3:** Secondary outcomes and adverse events

	Control (*n* = 11)	Hemoadsorption (*n* = 10)	Total (*n* = 21)	*p* value
*Secondary outcomes*				
Death within 14 days	7 (64)	7 (70)	14 (67)	1.00*
Death within 28 days	7 (64)	7 (70)	14 (67)	1.00*
ICU length of stay, days	4.5 (2.5, 5.8)	4.3 (2.8, 9.2)	4.5 (2.8, 5.8)	0.64^§^
CRP at day 2, mg/L^×^	207 (157, 249)	206 (129, 215)	206 (150, 221)	0.78^§^
Acute Kidney Injury^£^	7 (64)	9 (90)	16 (76)	0.31*
50% decrease in norepinephrine dose within 24 h	6 (54)	0 (0)	6 (28)	0.01*
50% decrease in norepinephrine dose within 48 h^×^	6 (60)	2 (22)	8 (42)	0.17*
Shock reversal within 24 h	5 (45)	1 (10)	6 (29)	0.15*
Serum lactate at 24 h, mmol/L	1 (1, 3)	2 (2, 5)	2 (1, 4)	0.20^§^
Serum lactate at 48 h, mmol/L^×^	1 (1, 1)	1 (1, 2)	1 (1, 2)	0.35^§^
Norepinephrine at 24 h, µg/min	1 (0, 32)	18 (16, 33)	16 (1, 32)	0.09^§^
Norepinephrine at 48 h, µg/min^×^	2 (0, 8)	11 (2, 26)	4 (0, 26)	0.22^§^
Change in SOFA score at day 1	1 (0, 2)	2 (1, 3)	1 (1, 3)	0.17^§^
Change in SOFA score at day 2^×^	− 1 (− 3, 2)	3 (1, 4)	1 (− 1, 4)	0.02^§^
*Adverse events*				
RBC within 2 days^§§^, units	3	3	6	
Elevated aPTT**	2 (18)	5 (50)	7 (33)	0.18*
Thrombopenia^¤^ within 1 day	2 (18)	5 (50)	7 (33)	0.18*
Severe thrombopenia^¤¤^ within 1 day	0 (0)	0 (0)	0 (0)	
Thrombopenia within 2 days^×^	3 (30)	5 (56)	8 (42)	0.37*
Severe thrombopenia within 2 days^×^	1 (10)	3 (33)	4 (21)	0.30*
Study catheter-related infection	–	0 (0)	–	
Major bleeding event	1 (9)	1 (10)	2 (10)	1.00*

Data are presented as median (interquartile range—IQR) for continuous measures, and *n* (%) for categorical measures

CRP C-reactive protein, ICU intensive care unit, RBC red blood cells, SOFA sequential organ failure assessment

^×^Only survivors at day 2 were included (*n* = 10 in control group and *n* = 9 in hemoadsorption group)

*Fisher’s exact test. §: Wilcoxon rank-sum test

^£^AKI is defined as any of the following: increase in serum creatinine by × 0.3 mg/dl within 48 h; or increase in serum creatinine to × 1.5 times baseline, which is known or presumed to have occurred within the prior 7 days; or urine volume < 0.5 ml/kg/h for 6 h

**Elevated aPTT ≥ 1.5 times the value at HA initiation in intervention group and/or aPTT ≥ 1.5 times the value at ICU admission, within 2 days after randomization. ^§§^Total count for the population. ^¤^Thrombopenia < 150G/L and thrombocytes at randomization > 150G/l. ^¤¤^Thrombopenia < 100G/L and thrombocytes at randomization > 150G/l

## Discussion

### Key findings

We conducted a pilot study to evaluate the safety and feasibility of hemoadsorption in cardiac arrest survivors at risk of PCAS. We found that the intervention could easily be implemented and was well tolerated. In particular, the regional anticoagulation protocol based on heparin and protamine infusions appeared safe and efficient to maintain the circuit patency for 24 h. We observed a trend for more thrombopenia in the treatment group but this did not lead to an increased need for transfusions. The intervention appeared to demonstrate some efficacy since it was associated with a trend toward a higher reduction in IL-6 plasma levels and other cytokines. The magnitude of this reduction was higher in patients with very elevated cytokine plasma levels at randomization.

### Comparisons with previous studies

To the best of our knowledge, this is the first randomized trial to evaluate the safety and feasibility of hemoadsorption in CA survivors without ECMO. Two other studies have evaluated such therapy in patients with ECMO. In the first, which was conducted in Germany, 50 CA survivors under ECMO were randomly allocated to receive 72 h of HA with CytoSorb^®^ or standard of care. Median IL-6 value at 72 h was not statistically different between groups, even after adjusting for baseline IL-6 level. Compared to our trial and despite higher baseline IL-6 plasma levels and longer duration of HA, the intervention was associated with a smaller absolute change in IL-6 within 72 h [19]. Also, the intervention was associated with a nonsignificant increase in mortality at 30 days (86% vs 58% in the control group, *p* = 0.08). In an observational study comparing 24 CA survivors treated with HA for 72 h with 48 matched historical controls, HA with CytoSorb^®^ was associated with higher mortality (83 vs 65%, *p* = 0.011)[20]. IL-6 plasma levels were not available. Our results on mortality do not confirm this trend for an increase in mortality in the HA group. Given the small sample sizes, the probability that these mortality differences can be attributed to a type 1 error appears to be high. However, this uncertainty regarding mortality calls for further prospective randomized trials adequately powered and including homogeneous and carefully selected population.

### Implications for clinicians and policymakers

The median absolute reduction in IL-6 at 48 h was small and not statistically significant. However, previous studies found that even small differences in IL-6 blood levels such as 100 pg/ml versus 200 pg/ml could make a difference in the subsequent development of organ failure [8, 21]. On the other hand, HA also appeared to be associated with a trend toward greater reduction in anti-inflammatory cytokines (IL-4 and IL-10) levels, which could be questioned as their effect might be necessary to counterbalance pro-inflammatory effects. However, numerous studies have observed an association between high levels of IL-10 after PCAS and mortality [11, 22]. Similarly, the observed slower reduction in vasopressors requirements in the hemoadsorption group is unexplained and might correspond to a type 1 error. Larger studies are required to confirm or refute these preliminary observations. Such trials should evaluate the impact of cytokines removal on patients’ centered outcomes such as mortality and, particularly in this context, neurological disabilities. Finally, thresholds of cytokines levels that should lead to hemoadsorption initiation as well as dose and duration of treatment still have to be determined to offer each patient the best therapy.

### Strengths and limitations

Besides its randomized design, this study has several strengths. Our efficacy criteria included serial measurements of a large panel of pro- and anti-inflammatory cytokines, at six different time points over 72 h post-randomization. IL-6 plasma measurements were highlighted in view of the amount of data supporting its role in PCAS development and potential interest as a therapeutic target to reduce myocardial injury in PCAS [10, 21, 23]. In addition, adverse events related to HA were collected and reported. This included bleeding events, thrombopenia, thrombosis and infections. We also provided safety data regarding the utilization of HA in a stand-alone modality with a heparin–protamine protocol. Controversy exists regarding the effects of hypothermia and rewarming rate on inflammation, which could have interfered with our results [24, 25]. However, temperature management was applied in a similar fashion to patients in both groups.

Certain limitations should also be reported. First, as a pilot study, it was not powered to detect a difference in mortality or in cytokines reduction. Therefore, the small sample size precludes any conclusions on biological and clinical outcomes. In addition, for this reason, we explored cytokines variations mainly through descriptive analysis and simple comparison tests. Our data enable power calculation for further studies, which would ideally include more statistical analyses such as mixed effects models accounting for time effects and baseline characteristics. Second, the randomization was imperfect and CA characteristics associated with worse outcomes (time-to-ROSC, out-of-hospital CA, non-shockable presenting rhythm) as well as certain comorbidities (chronic heart failure, hypertension, aspirin use and smoking) were unbalanced between groups. The exact impact of this imbalance is difficult to estimate as characteristics associated with poor prognosis were distributed between both groups (longer time-to-ROSC and more out-of hospital CA in HA group but more non-shockable rhythm and higher admission lactate in control group). Third, we did not collect neurological outcomes; however, HA may exert beneficial effects on neurological recovery. Previously, elevated IL-6 levels in cerebrospinal fluid (CSF) were found to be associated with poor neurological outcomes [10]. Because IL-6 is able to cross the blood–brain barrier, reduction in plasma levels with HA may also reduce CSF levels and improve neurological recovery. Fourth, HA was initiated almost 24 h after CA in our study. An earlier initiation could reduce the inflammatory mediator’s burden before subsequent organ damage occurs. However, even if some results are discordant, most studies reported a peak of IL-6 plasma levels at 24 h, suggesting that we initiated HA at the most striking time of inflammation [22, 24]. Compared to patients with sepsis, the onset of aggression is known in CA survivors and therefore future trials could compare different timings of initiation. The optimal dose and duration could also be questioned. We used only one single hemoadsorption cartridge, for a total maximum duration of 24 h, which may have been insufficient since only 29% of our patients reversed their shock within this timeframe. Because hemoadsorption may decrease over time, the use of more cartridges, each for a shorter duration could also be considered in future trials [26]. Fifth, we decided to exclude patients with an RRT indication and to perform HA exclusively in a stand-alone modality. This allowed us to rule out biases related to potential cytokine’s elimination by CRRT, as previously suggested [13]. However, more conventional utilization of HA (in line with CRRT) is likely to result in similar or increased cytokine clearance but would enable the use of regional citrate anticoagulation and thus increase HA safety. In addition, the use of HA in a stand-alone modality may expose patients to complications related to the extracorporeal circuit. Thus, thrombopenia is a common side effect of extracorporeal circuits use and our study did not allow us to distinguish between an event related to the circuit or to the HA treatment itself. Similarly, we observed a trend for more elevated aPTT in the HA group which was most probably due to heparin leakage into the systemic circulation than HA effect. Such adverse events are likely to be more limited when HA is combined with RRT since regional citrate anticoagulation can then be administered.

Finally, the study population was highly heterogeneous with respect to IL-6 plasma levels at randomization. Although we applied strict criteria to select the patients with the highest risk to develop PCAS (therefore excluding 30% of patients admitted after CA), IL-6 plasma levels were low (< 100 pg/mL) at randomization in half of our patients. Because hemoadsorption is a concentration-dependent process, its effect on these patients is likely to be negligible and might even “dilute” any potential effect observed in those with the highest levels. Including patients unlikely to benefit from the treatment by nature leads to erroneous conclusions while exposing them to potential adverse events. In the absence of cytokine measurements at the bedside, future research should focus on identifying predictors of elevated cytokines, such as biomarkers or clinical scores.

## Conclusions

In CA survivors at risk of PCAS without ECMO, early hemoadsorption with CytoSorb^®^ was safe and feasible. Compared with standard care, it was associated with a nonsignificant reduction in plasma cytokine levels. Any future trial evaluating HA should consider including a measure of IL-6 or another circulating cytokine in their inclusion criteria to enrich the population.

## Supplementary Information

**Additional file 1**. Protocol for regional circuit anticoagulation.

**Additional file 2**.  Technical characteristics of hemoperfusion treatments.

## Data Availability

The datasets used and/or analyzed during the current study are available from the corresponding author upon reasonable request.

## Abbreviations

aPTT: Activated partial thromboplastin time
CA: Cardiac arrest
ECMO: Extracorporeal membrane oxygenation
ELISA: Enzyme-linked immunosorbent assay
HA: Hemoadsorption
ICU: Intensive care unit
IFN: Interferon
IL: Interleukin
IQR: Interquartile range
PCAS: Post-cardiac arrest syndrome
ROSC: Return of spontaneous circulation
RRT: Renal replacement therapy
TNF: Tumor necrosis factor
