# Supplementary material for: Cytokine hemoadsorption with CytoSorb® in post-cardiac arrest syndrome, a pilot randomized controlled trial
Source: Crit Care. 2023 Jan 23;27:36. doi: 10.1186/s13054-023-04323-x (PMC9869834; doi:10.1186/s13054-023-04323-x)
Supplement: Supplementary file 1 — Additional file 1. Protocol for regional circuit anticoagulation. [file 13054_2023_4323_MOESM1_ESM.docx]

**Additional file 1**

**Protocol for regional circuit anticoagulation**

**Standard Operating Procedure for Heparin-Protamin Regional Anticoagulation**

- Prior to therapy initiation: measurement of baseline aPTT which is the patient systemic aPTT activity
- Heparin:
  - Administered directly in the afferent line of the extra-corporeal circuit
  - Initial dose: 1000 UI/h
  - Adapted according to “circuit” aPTT (measured at the post-filter level) targeting an aPTT 1.5 to 2x baseline
- Protamine:
  - Administered in the return line of the extra-corporeal circuit
  - Initial dose: 10 mg/h
  - Adapted according to “patient” aPTT (measured through arterial or central line) targeting a normal (< 40 secondes) aPTT.
- Both “circuit” and “patient” aPTT are measured every 6 hours during the therapy. Systemic aPTT is also measured 6 and 12 hours after therapy is resumed.
