# Supplementary material for: Cytokine hemoadsorption with CytoSorb® in post-cardiac arrest syndrome, a pilot randomized controlled trial
Source: Crit Care. 2023 Jan 23;27:36. doi: 10.1186/s13054-023-04323-x (PMC9869834; doi:10.1186/s13054-023-04323-x)
Supplement: Supplementary file 2 — Additional file 2. Technical characteristics of hemoperfusion treatments. [file 13054_2023_4323_MOESM2_ESM.docx]

**Table. Technical characteristics of hemoperfusion treatments ( n=10)**

| Averaged blood flow, ml/min* | 300 (271-300) |
| --- | --- |
| Duration of session, hours | 21.25 (14.3-23.6) |
| Total amount of blood treated, liters* | 375 (360-432) |
| Data are expressed as median (interquartile range)  *Blood flow was unavailable for one patient, data are for 9 patients | |
